# Biophysical and structural insights into AAV genome ejection

**DOI:** 10.1128/jvi.00899-24

**Published:** 2025-02-05

**Authors:** Keely Gliwa, Joshua Hull, Austin Kansol, Victoria Zembruski, Renuk Lakshmanan, Mario Mietzsch, Paul Chipman, Antonette Bennett, Robert McKenna

**Affiliations:** 1 Department of Biochemistry and Molecular Biology, University of Florida 166768 , Gainesville, Florida, USA; 2 ICBR Electron Microscopy Core Facility, University of Florida 3463 , Gainesville, Florida, USA; Cornell University Baker Institute for Animal Health, Ithaca, New York, USA

**Keywords:** genome, capsid, structure, stability, cryo-EM

## Abstract

Recombinant adeno-associated virus (rAAV) is comprised of non-enveloped capsids that can package a therapeutic transgene and are currently being developed and utilized as gene therapy vectors. The therapeutic efficiency of rAAV is dependent on successful cytoplasmic trafficking and transgene delivery to the nucleus. It is hypothesized that an increased understanding of the effects of the cellular environment and biophysical properties of the capsid as it traffics to the nucleus could provide insight to improve vector efficiency. The AAV capsid is exposed to increasing [H^+^] during endo-lysosomal trafficking. Exposure to low pH facilitates the externalization of the viral protein 1 unique region (VP1u). This VP1u contains a phospholipase A2 domain required for endosomal escape and nuclear localization signals that facilitate nuclear targeting and entry. The viral genome is released either after total capsid disassembly or via a concerted DNA ejection mechanism in the nucleus. This study presents the characterization of genome ejection (GE) for two diverse serotypes, AAV2 and AAV5, using temperature. The temperature required to disassemble the virus capsid (T_M_) is significantly higher than the temperature required to expose the transgene (T_E_) for both serotypes. This was verified by quantitative PCR (qPCR) and transmission electron microscopy. Additionally, the absence of VP1/VP2 in the capsids and a decrease in pH increase the temperature of GE. Furthermore, cryo-electron microscopy structures of the AAV5 capsid pre- and post-GE reveal dynamics at the twofold, threefold, and fivefold regions of the capsid interior consistent with a concerted egress of the viral genome.

**IMPORTANCE:**

The development of recombinant adeno-associated virus (rAAV) capsids has grown rapidly in recent years, with five of the eight established therapeutics gaining approval in the past 2 years alone. Clinical progression with AAV2 and AAV5 represents a growing need to further characterize the molecular biology of these viruses. The goal of AAV-based gene therapy is to treat monogenic disorders with a vector-delivered transgene to provide wild-type protein function. A better understanding of the dynamics and conditions enabling transgene release may improve therapeutic efficiency. In addition to their clinical importance, AAV2 and 5 were chosen in this study for their diverse antigenic and biophysical properties compared to more closely related serotypes. Characterization of a shared genome ejection process may imply a conserved mechanism for all rAAV therapies.

## INTRODUCTION

**Fig 1:**
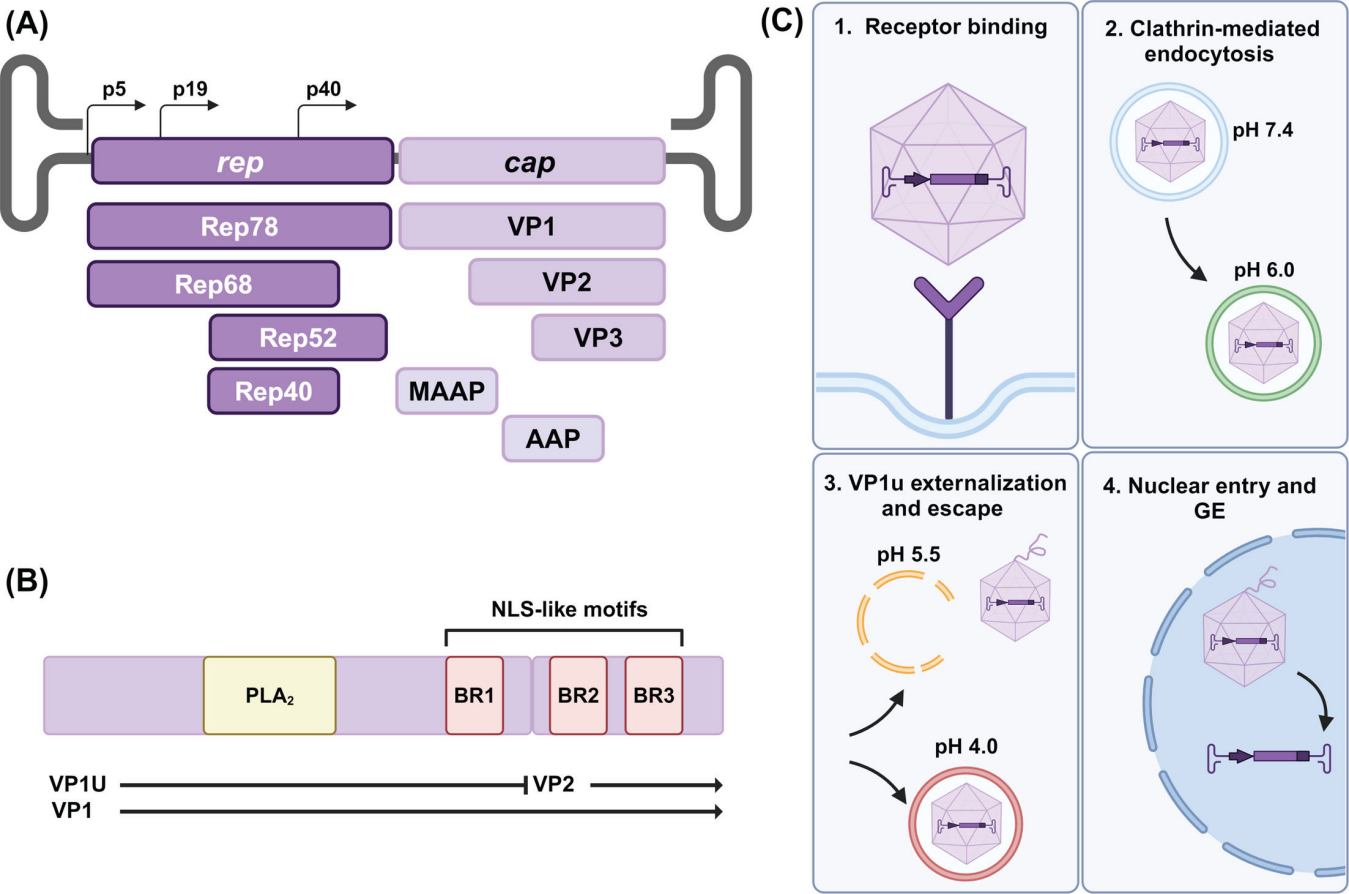
Cartoon depiction of the AAV genome organization and key steps of the viral life cycle. (**A**) The AAV genome with ORFs, *rep* and *cap,* and the encoded proteins are shown below the respective ORFs. (**B**) Functional regions of the VP1 unique region and VP2 including the phospholipase domain (PLA2) and basic regions (1-3), respectively. (**C**) Key steps of the AAV viral life cycle.

Adeno-associated virus (AAV) are small, non-pathogenic, single-stranded DNA viruses classified in the *Parvoviridae* family, the *Parvovirinae* subfamily, and the *Dependoparvovirus* genus (1, 2). Characteristic of the genus, AAV depends on a helper virus for successful replication (3, 4). The AAVs package a ~4.7 kilobase (kb) genome in a non-enveloped, icosahedral (T = 1) capsid with a diameter of ~260 Å. The genome contains two main open reading frames (ORFs), *rep* and *cap*, flanked by two inverted terminal repeats (ITRs) (Fig. 1A). *Rep* encodes four non-structural proteins: Rep78, Rep68, Rep52, and Rep40. These are required for viral DNA replication and packaging (5). The *cap* ORF encodes three overlapping structural viral proteins (VPs): VP1, VP2, and VP3 (6). The assembled capsid is comprised of 60 monomers in an approximate ratio of 1:1:10 of VP1:VP2:VP3 (7, 8). The entire VP3 sequence is shared among the VPs and termed the VP3 common region. VP3 is responsible for assembling the body of the capsid, which functions to transport and protect the viral genome. The VP2 has an extended N-termini shared with VP1 termed the VP1/VP2 common region. In this common region, a series of basic regions have been identified to function as nuclear localization signals (9, 10). VP1 is the largest of the VPs and has a unique N-terminus (VP1u or VP1 unique region). The VP1u contains a phospholipase A2 (PLA2) domain essential for endo-lysosomal escape during viral trafficking (Fig. 1B) (10, 11). Two additional proteins have been identified from a +1 frameshift ORF in the *cap* gene: assembly activating protein (AAP) and membrane-associated accessory protein (MAAP). AAP and MAAP have been implicated in capsid assembly and viral egress, respectively (12–14).

The AAV capsid has multiple functions in the viral life cycle, including cellular entry initiated through receptor and co-receptor binding, and cellular trafficking following clathrin-mediated endocytosis (15). During trafficking the capsid experiences increasing acidification with a transition from the early (pH 6.0) to late endosome (pH 5.5) and lysosome (pH 4.0) (Fig. 1C) (16). This change in local pH is thought to contribute to conformational changes that induce VP1u externalization (Fig. 1C) (17). The VP1u externalization and resulting PLA2 activity are essential for successful trafficking, and capsids without surface-accessible VP1u do not traffic to the nucleus. It is currently believed that VP1u externalizes through the fivefold symmetry axis, though additional evidence suggests a role for the twofold in VP1u activity (18, 19). There are two currently proposed mechanisms for nuclear localization after cytoplasmic trafficking: traditional entry via the nuclear pore complex or alternative entry via transient holes in the nuclear envelope (20). Conjointly, it is currently believed that intact AAV capsids enter the nucleus prior to genome release (Fig. 1C) (16, 21–23). The mechanism for genome ejection (GE) is poorly understood, although this represents a critical step in gene delivery to the nucleus for genome replication and transcription. After DNA replication, the genome is packaged into assembled, or pre-formed capsids (24). This is also believed to occur via a pore at the fivefold axis (18). Nucleotides have been modeled near the threefold axis in a DNA binding pocket, and several AAV2 threefold variants have a severely reduced genome packaging phenotype (25, 26). A defective genome packaging or retention phenotype is also associated with several fivefold variants (18, 27). This study will probe the GE process to better characterize fundamental AAV biology and potentially inform the development of improved viral vectors.

To date, there have been eight AAV-based approved gene therapeutics with an additional 100+ clinical trials listed on clincialtrials.gov., majority of the currently approved therapies utilize serotypes AAV2 and AAV5. In addition to their clinical relevance, AAV2 and AAV5 represent diverse antigenic, structural, and biophysical properties compared to the 13 recognized AAV serotypes. The phylogenetic relationship among the AAV serotypes is determined through sequence homology of the *cap* gene, ranging from 52% to 99% for VP1 (28). Similarly, structural homology ranges from 62% to 99% (28). AAV5 is the most evolutionarily divergent of the AAV serotypes (29). Consequently, AAV2 and AAV5 share only 57% VP1 sequence identity, and 69% structural identity (28, 29). As a result, AAV2 and AAV5 are ideal candidate serotypes to investigate a conserved or divergent mechanism for GE. In addition, these serotypes also represent the lowest and highest capsid melting temperatures (T_M_) for the AAVs. In phosphate-buffered saline (PBS), AAV2 has the lowest T_M_ at 68°C and AAV5 has the highest T_M_ at 89°C (30). This study takes advantage of AAV’s thermostability, and modulating temperature to induce capsid conformational changes to simulate GE.

## RESULTS AND DISCUSSION

### Purification of genome containing AAV2 and AAV5 capsids

**Fig 2:**
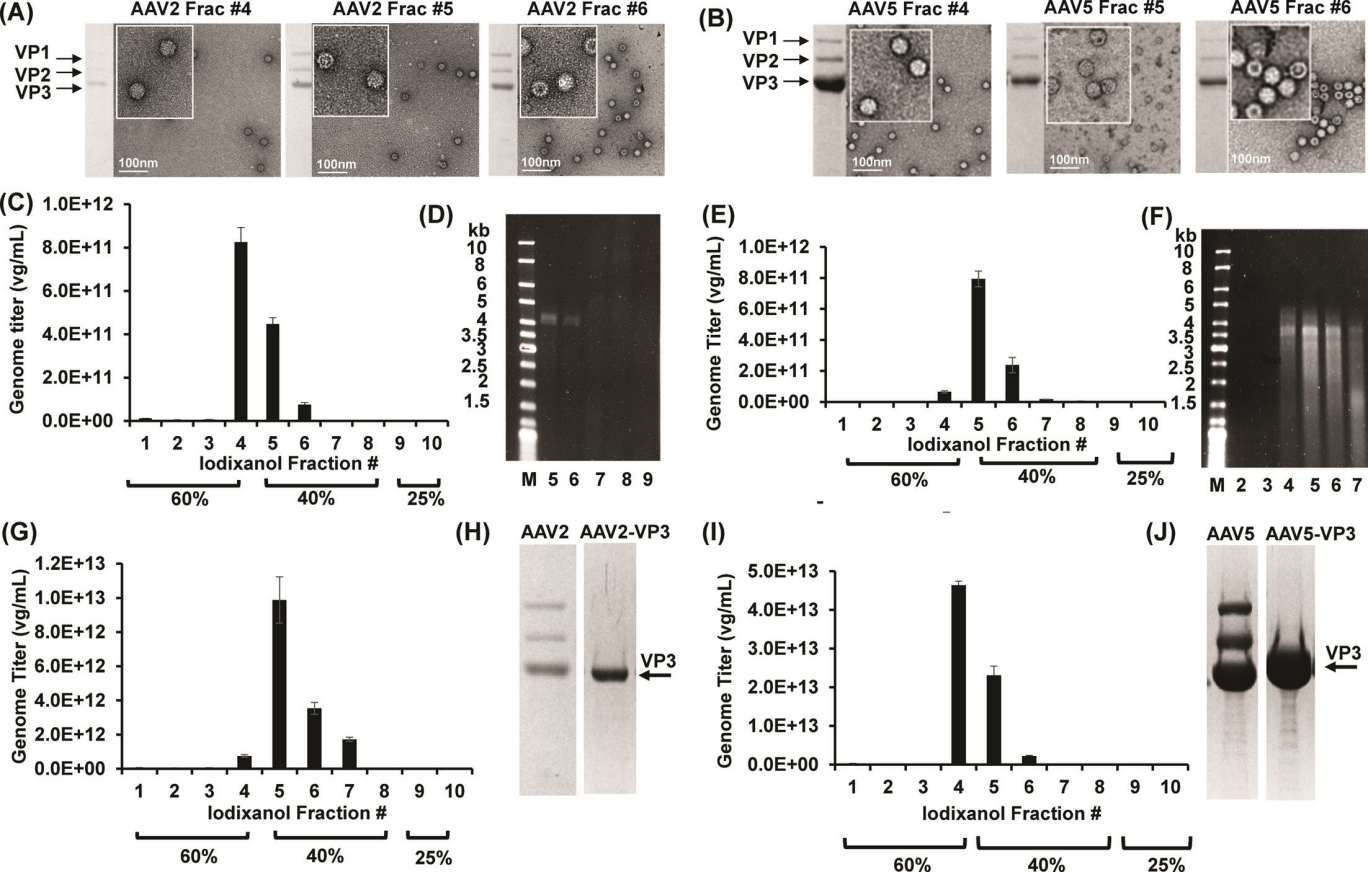
Purification of AAV2-fulls, AAV5-fulls, and VP3-fulls (stock samples). (**A and B**) SDS-PAGE (left) and negative stain EM (right) for AAV2 and AAV5 iodixanol fractions. (**C**) qPCR and (**D**) alkaline gel of AAV2 iodixanol fractions. (**E**) qPCR and (**F**) alkaline gel of AAV5 iodixanol fractions. (**G**) qPCR of AAV2-VP3 iodixanol fractions and (**H**) SDS-PAGE of purified AAV2-VP3. (**I**) qPCR of AAV5-VP3 iodixanol fractions and (**J**) SDS-PAGE of purified AAV5-VP3.

The AAVs were purified using iodixanol density gradient centrifugation as previously described (31). Ten 1 mL fractions separated by iodixanol step gradients, at fraction 4–5 (40%/60% interface), fraction 6–8 (40%), and fraction 9–13 (25%) were enriched with AAV-fulls (DNA genome packaged), AAV-fulls/AAV-empties mixture, and AAV-empties (Fig. 2). These fractions were confirmed by negative stain electron microscopy (EM), quantitative polymerase chain reaction (qPCR), and alkaline gel electrophoresis (Fig. 2). Full capsids are not stain-penetrated and thus appear solid white in the micrographs (Fig. 2A and B). For both AAV2 and AAV5, stain penetration is visible in fractions 6 and 7, indicative of capsids without fully packaged genome, or empties lacking any packaged DNA. This observation was corroborated by qPCR data, suggesting peak genome titer between fractions 4 and 5 (Fig. 2C and E). Similarly, the alkaline gels show a decrease in band intensity as the fraction number increases, suggesting fewer full capsids (Fig. 2D and F). To avoid purification of empty capsids, fractions 6 (40% iodixanol fraction) and greater were excluded from further purification, and the purified AAV-fulls were utilized for the heat-treated negative stain EM and cryo-EM structure determination.

### Production and purification of VP3 variants

Variants of AAV2 and AAV5 capsids (AAV2-VP3 and AAV5-VP3) were generated via site-directed mutagenesis. The VP1 and VP2 start codons were mutated to leucine and premature stop codons, respectively. The AAV2-VP3 and AAV5-VP3 were produced and purified as described for wild-type (wt) AAV2 and AAV5. Similarly, full capsids were detected, and genome titer peaks around fractions 4 and 5 according to qPCR data (Fig. 2G and I). Final purification results after column chromatography and centrifugal filter concentration are shown on SDS-PAGE (Fig. 2H and J). SDS-PAGE analysis confirms the purity and identity of the wt and variant capsids. A single band is seen for the variant constructs at the appropriate molecular weight of VP3. Three bands indicating VP1, VP2, and VP3 are shown for the respective wt AAV. The AAV2 genome titer was 83.4% less than that of AAV2-VP3. Similarly, AAV5 genome titer was 76.3% less than that of AAV5-VP3. It has been previously demonstrated that genome packaging can be optimized by creating hybrids of the *rep* gene (32). Importantly, variant and wt AAV capsids in this study contain the same ITRs and rep ORF, expressing the same non-structural proteins associated with replication and genome packaging (27, 33). The ability of wt capsids to efficiently package the transgene may be restricted by the internal occupancy of VP1, VP2, and the VP3-N terminus. Both VP1, VP2 and the viral genome utilize the interior of the virus capsid and may interact in this shared space. It is not known if VP1u externalization affects the retention or ejection of the viral genome. However, it has been shown that the genome’s size plays a role in the rate of ejection (34). The utilization of VP3 capsid variants provides additional insights into the interaction of the minor capsid VPs and GE.

### Genome ejection is independent of capsid disassembly

The physiological conditions and capsid dynamics required for AAV capsids to release their genome have not been previously determined. As a result, the mechanism for AAV uncoating is poorly understood. There are two potential possibilities for the viral genome to become accessible: (i) capsid disassembly or (ii) a concerted release from intact capsids. Here it is hypothesized that GE occurs in a controlled step following nuclear entry, governed by similar capsid conformational changes that facilitate genome packaging and VP1u externalization. During trafficking, the structural viral proteins are protective, shielding the genome from degradation in the cytoplasm. The capsid must maintain enough stability to do this efficiently. Yet, dynamic structural transitions are required for the successful completion of the viral life cycle and therapeutic gene expression. This “metastable” characteristic facilitates receptor binding, endo-lysosomal escape, genome packaging, etc. (35). This study investigates if genome release occurs in a similar fashion to packaging, mediated by intact capsid dynamics.

Previous work has shown that temperature can trigger genome release from AAV particles (36). The capsid is presumably destabilized during genome release *in vivo,* and the use of heat allows for controlled capsid reorganization and disruption *in vitro*. The goal of these studies was to quantify a decrease in the packaged genome with increasing temperature and to determine if there is a distinction between capsid melting temperature (T_M_) and genome ejection temperature (T_E_). The T_M_ is defined as the temperature at which 50% of the capsids are denatured and this value is determined by differential scanning fluorimetry (DSF). The T_E_ is defined as the temperature at which there is a loss of 50% of the starting genome titer and this value is obtained by qPCR, all T_E_ values are ±2°C.

**Fig 3:**
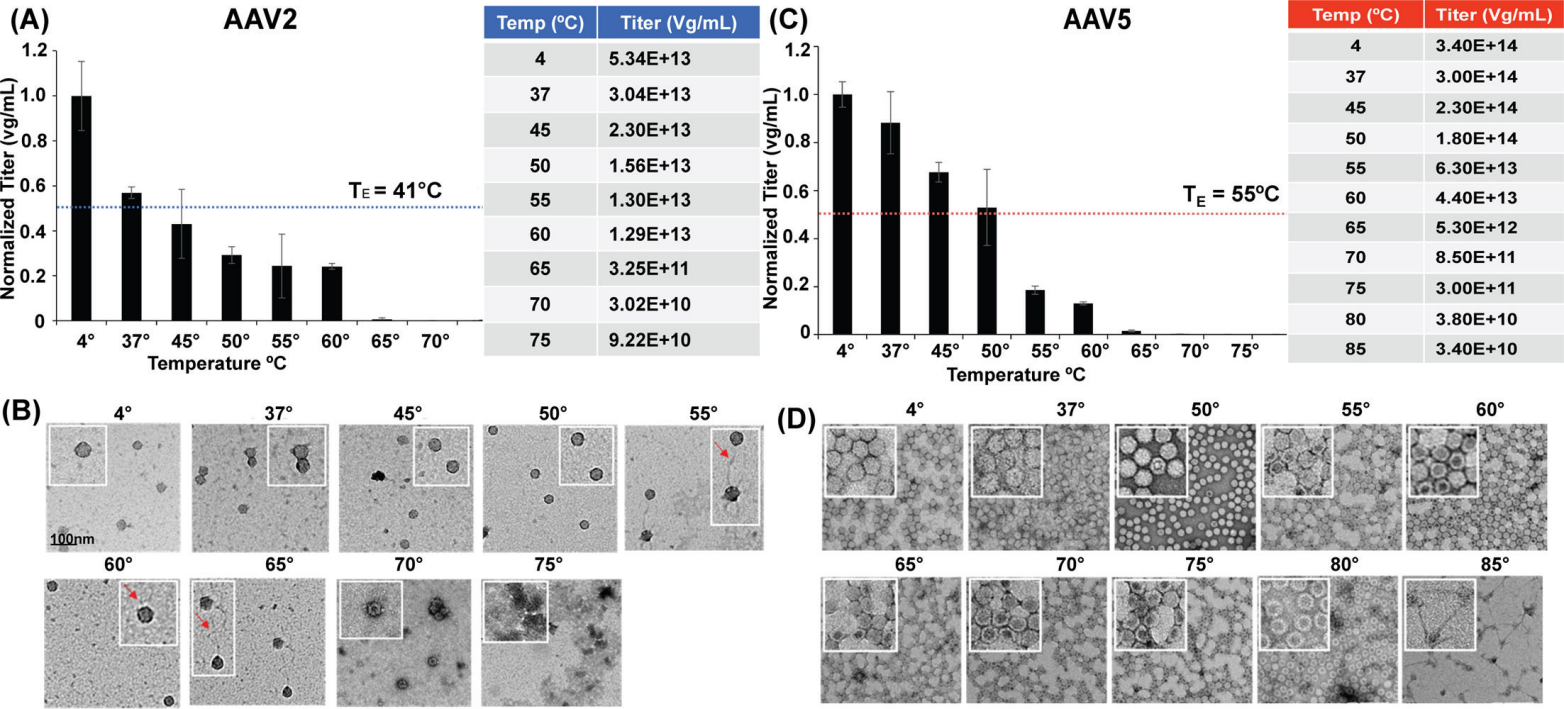
Genome ejection from intact AAV2-fulls and AAV5-fulls (stock samples). (**A**) Quantification of the ejected genome after temperature incubation and DNase I treatment of AAV2 capsids and (**B**) corresponding negative stain EMs without DNase I treatment. (**C**) Quantification of the ejected genome after temperature incubation and DNase I treatment of AAV5 capsids and (**D**) corresponding negative stain EMs without DNase I treatment. T_E_ is defined as the temperature at which there is 50% of the starting genome titer.

AAV2 capsid has been previously established as the least thermally stable of the analyzed AAVs. Following heat treatment and DNase I digestion, 50% of the initial genomes were released between 37°C and 45°C, with a T_E_ of 41°C (Fig. 3A). Between 50°C and 60°C, a fivefold reduction from the starting titer was observed (Fig. 3A). This was coupled with extensive changes in capsid morphology based on negative stain EM done without DNase I digestion. At 50°C, string-like material is first observed to be emerging from the AAV2 capsid, as indicated by red arrows (Fig. 3B). These “strings” are also seen at 55°C and 60°C, preceding the greatest drop in genome titer (from 60°C to 65°C), according to qPCR. The emergent strings are likely strands of DNA ejected from the interior of intact capsids. Most capsids are stain penetrated at 65°C, indicative of genome loss, and completely denatured at 70°C (Fig. 3B). Morphology from the EMs is consistent with the previously reported T_M_ of AAV2 in PBS buffer at approximately 65°C (30).

In contrast to AAV2, the AAV5 capsids released approximately 50% of the packaged genome by 55°C (Fig. 3C). This corresponded to AAV2 and AAV5 capsid structural changes observed by negative stain EM. Specifically, the increase in stain penetration, indicative of the formation of empty capsids. Most capsids appear to be empty at 80°C, with observable string-like material extruded at 85°C (Fig. 3D). It is likely that emergent strings are not observed until just before denaturation due to the dense population of AAV5 capsids compared to the AAV2 sample. The qPCR data imply that the genome is released prior to this temperature (Fig. 3C). Previously, the T_M_ values have been determined and are known to be pH, buffer, and AAV serotype-specific (30). Importantly, a loss in the packaged genome is observed prior to the capsid T_M_ in both AAV2 and AAV5 (Fig. 3). This indicates that GE is not due to capsid disassembly in this assay. Although AAV5 has a T_M_ approximately 20°C–25°C greater than AAV2 in PBS buffer (89°C and 65°C, respectively), both serotypes have released most of their genome by 55°C. This study has developed a method to investigate AAV genome ejection independent of total capsid disassembly *in vitro*. While these temperatures are not the physiological trigger for genome release, GE is believed to occur from intact capsids (16, 21–23, 37), so preserving this detail more closely enables possible *in vivo* dynamics. If genome release was controlled solely by temperature, one might expect distinct profiles for AAV2 and AAV5 given their distinct thermostabilities. This suggests that uncoating is independent of capsid disassembly *in vitro* and therefore a shared mechanism for both capsids.

### Genome ejection is pH-sensitive and VP-dependent

**Fig 4:**
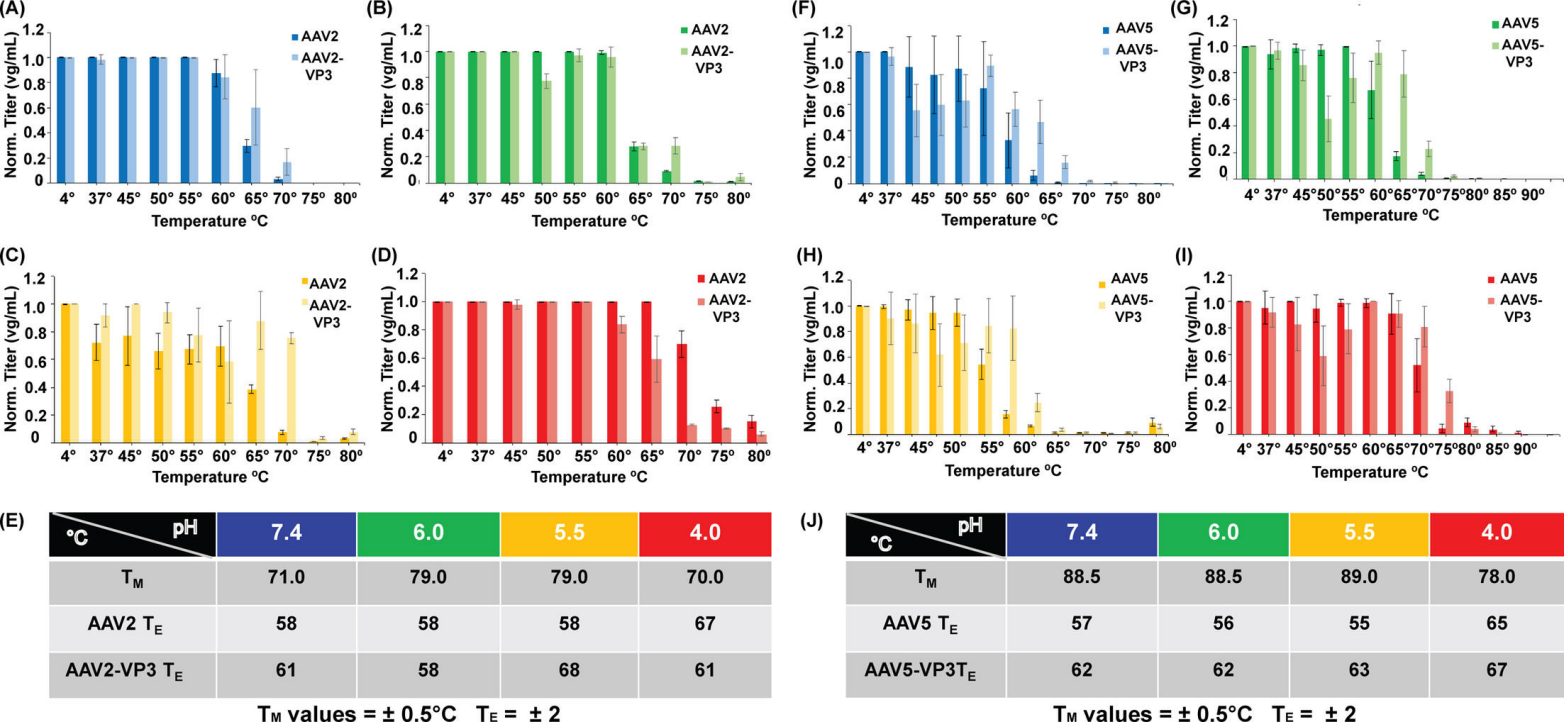
Genome ejection of VP3 variants at endo-lysosomal pH. Quantification of the ejected genome after temperature incubation of AAV2 and AAV2 VP3 capsids at (**A**) pH 7.4, (**B**) pH 6.0, (**C**) pH 5.5, (**D**) pH 4.0, and (**E**) table of T_M_ and T_E_ values. Quantification of the ejected genome after temperature incubation of AAV5 and AAV5 VP3 capsids at (**F**) pH 7.4, (**G**) pH 6.0, (**H**) pH 5.5, (**I**) pH 4.0, and (**J**) table of T_M_ and T_E_ values.

During cellular trafficking, AAV capsids experience increasing acidification in the endo-lysosomal pathway. Given the essential role of acidification in the viral life cycle, we performed the GE assay at four physiologically relevant pHs: 7.4, 6.0, 5.5, and 4.0. The T_M_ of AAV2 has been described to be the greatest at intermediate pHs (38). This trend was observed in the DSF analysis (Fig. 4E). At pH 4.0 and 7.4, the T_M_ is 70°C and 71°C, respectively. At the intermediate pHs (6.0 and 5.5), T_M_ is increased 8°C–10°C (Fig. 4E). It has been suggested that stabilization of the AAV capsid may be required to maintain capsid integrity while facilitating VP1u externalization (31). In contrast to AAV2 capsids, AAV5 was reported to demonstrate a consistent decrease in thermostability with increasing acidity (38). This was observed in the DSF analysis, with AAV5 capsids the least thermostable at pH 4.0 at 78°C (Fig. 4J). AAV5 T_M_ is within ±0.5°C across pH 7.4, 6.0, and 5.5 at approximately 88°C (Fig. 4J).

Relatedly, previous studies on heat treatment of MVM DNA-containing particles (at 45°C) prior to capsid disassembly (at 80°C) resulted in exposure of VP1u and increased accessibility of the viral genome (39). The authors infer that the conformational changes of VP1 dynamics also enable DNA release. A similar investigation for B19V found that nearly 90% of intact capsids released their genome by 60°C (40). Genome release by B19V capsids can also be triggered by low pH alone (40). This is unique to B19V, as its VP1u is permanently externalized after receptor binding (41). While low pH alone is insufficient to trigger VP1u exposure or genome loss in AAV particles, it has been shown to impact the secondary structure of VP1u and negatively affect vector transduction efficiency (17, 38). Therefore, this study investigates if VP1u externalization is a priming event for GE, and or if the necessary capsid conformational changes are shared for the two processes.

To investigate the role of VP1 and VP2 on AAV GE, the GE assay was performed using the previously described AAV-VP3 constructs. If the minor capsid VPs play a role in GE, then there should be a difference in T_E_ when using an AAV-VP3 construct. Importantly, changes in the T_E_ cannot be attributed to a change in capsid thermostability, given that VP3 is the sole determinant of capsid T_M_ (30). The T_E_ for AAV-VP3 is shown in the lighter-colored line for pH 7.4 (blue), 6.0 (green), 5.5 (yellow), and 4.0 (red) (Fig. 4). At all pHs, AAV5-VP3 capsids require higher temperatures to release their genome compared to wt AAV5 capsids (Fig. 4F through I). Similarly, AAV2-VP3 capsids also have a delayed profile of genome release compared to AAV2 (Fig. 4A through D), with an exception at pH 4.0. Interestingly, the trend is reversed at low pH in this serotype. Rather, AAV2 capsids retain genome at higher temperatures than AAV2-VP3 at pH 4.0 (Fig. 4D). For all constructs, there is genome loss prior to disassembly (Fig. 4E and J). It is important to note that while AAV2 T_M_ is 8°C–18°C lower than AAV5, the corresponding T_E_ values are within 3°C. This suggests a potentially conserved mechanism of GE for both AAV2 and AAV5.

### Structural characterization of AAV5 capsids pre- and post-genome ejection

The AAV capsid is a T = 1 icosahedron with twofold, threefold, and fivefold symmetry axes. There is a depression at the twofold axis, protrusions surrounding the threefold axis, and a channel at the pore of the fivefold axis. The relationship between these capsid features and viral dynamics has been illustrated for AAV capsid assembly, packaging, and infectivity. For example, amino acid substitutions of conserved residues surrounding the fivefold pore resulted in reduced genome packaging and infectivity of AAV2 capsids (18). Reduced infectivity was generally attributed to defective VP1u exposure, preventing accessibility to the PLA2 domain essential for endo-lysosomal escape (18). As a result, the current theory is that AAVs utilize the fivefold pore for both genome packaging and VP1u externalization. Though threefold variants were also observed to have defective genome packaging, this observation has been suggested to result from the loss of capsid stability (25). In addition, the twofold axis has also been implicated in VP1u dynamics. Chemical crosslinking of tyrosine residues along the twofold axis reduces cell transduction by hindering VP1u externalization (19). Low-resolution cryo-EM analysis has supported this potential relationship, with observed density under the twofold, described as globules, attributed to the N-termini of VP1 and/or VP2 (42), though confirmation at atomic resolution is needed. Interestingly, the movement of this density from the inner to outer capsid surface was specific to genome-containing particles, suggesting a relationship between packaged DNA and VP dynamics (42). Collectively, there is growing evidence that the axes of symmetry play multiple functional roles in the viral life cycle. To further investigate the structural dynamics of GE, cryo-EM was used to visualize the capsids pre- and post-genome release. To generate these populations, purified AAV5 capsids were incubated for 30 min at 4°C, 55°C, and 80°C prior to vitrification.

**Fig 5:**
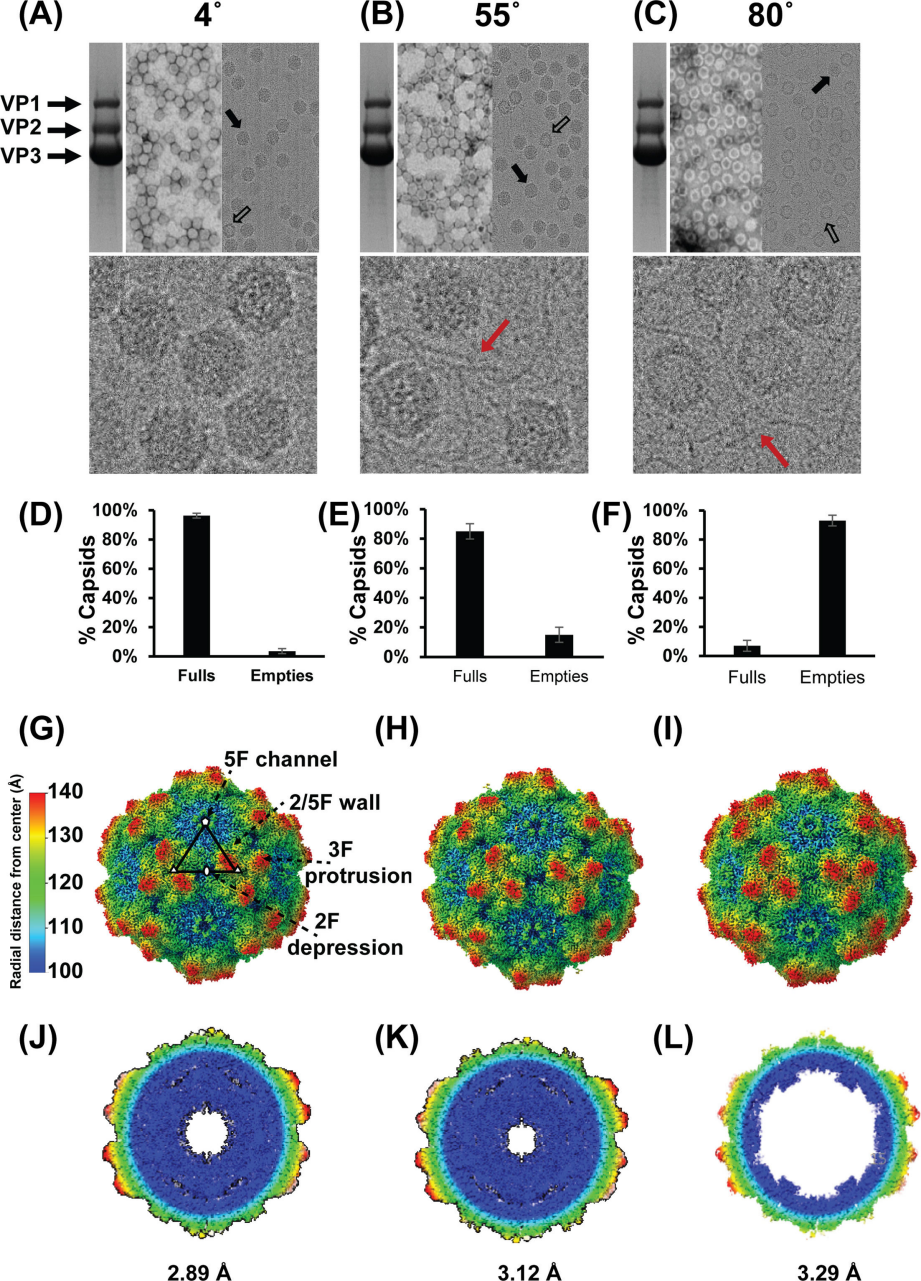
Structural characterization of AAV5-fulls (stock sample) pre- and post-genome ejection. (**A–C**) SDS-PAGE (left), negative stain EM (center), and cryo-EM micrographs (right) at 4°C, 55°C, and 80°C, respectively. (**D–F**) Quantification of full and empty capsids based on cryo-EM micrographs at 4°C, 55°C, and 80°C. (**G–I**) 3D image reconstruction of AAV5 capsids at 4°C, 55°C, and 80°C. (**J–L**) Cross section of AAV5 capsids at 4°C, 55°C, and 80°C.

Micrographs of AAV5 at 4°C, 55°C, and 80°C reveal the usual capsid morphology with no indication of disassembled capsids (Fig. 5A through C). Quantification of full versus empty capsids is verified using the cryo-EM micrographs (Fig. 5D through F). The major observed difference among the micrographs is the increasing percentage of empty capsids seen at higher temperatures. At 4°C, most capsids, ~96%, contain packaged genome (Fig. 5D). This is slightly reduced at 55°C to ~85% (Fig. 5E), and drastically reduced at 80°C to ~7% (Fig. 5F). The micrographs at 80°C show string-like material, likely DNA ejected from the full capsids to produce a large number of empty capsids. The predominant capsid type at each temperature was used for final 3D image reconstruction. Three-dimensional reconstruction was conducted using the cryoSPARC software package (43). All 2D classes of empty particles at 4°C and 55°C and full particles at 80°C were excluded from the reconstruction process.

**Fig 6:**
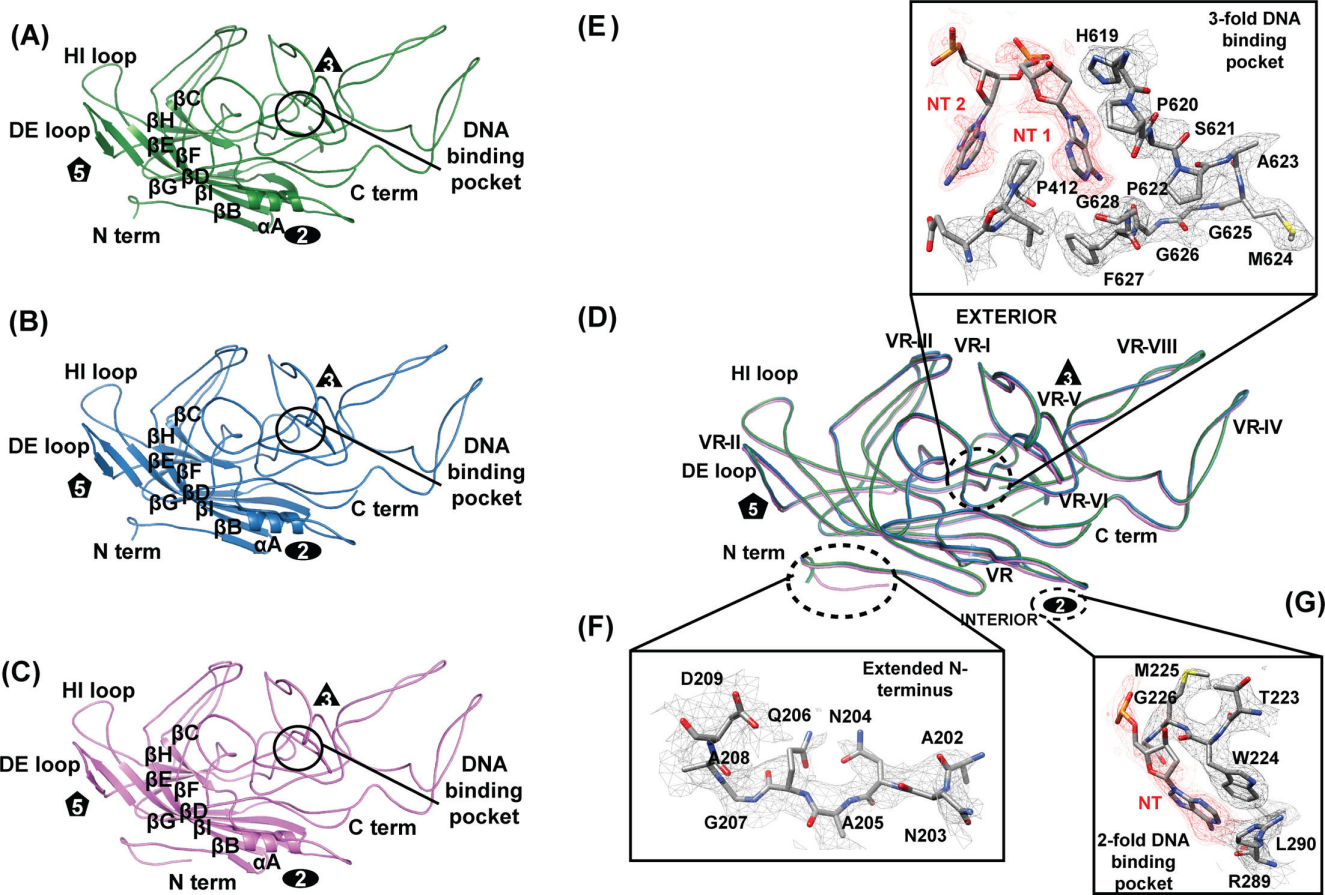
Key structural features of the AAV5 capsid. Monomer representations with labeled secondary structure, symmetry axes, surface loops, and the C- and N-terminal domains (CTD, NTD) at (**A**) 4°C, (**B**) 55°C, and (**C**) 80°C shown in green, blue, and pink, respectively, and (**D**) superimposed without secondary structure. (**E**) Zoomed view of the threefold DNA binding pocket with nucleotide density at contour level 2σ (red) and level 1σ (pink) and associated capsid residues at level 3σ (black) at 4°C shown in the upper right corner. (**F**) Zoomed view of the extended N-terminus in electron density map at contour level 1σ with labeled residues at 80°C shown in the bottom left corner. (**G**) Zoomed view of the twofold DNA binding pocket with fit nucleotide density at contour level 2σ (red) and level 1σ (pink) and associated capsid residues at level 3σ (black)at 4°C shown in bottom right corner.

The cryo-EM structures of the AAV5 capsids at 4°C, 55°C, and 80°C were determined to be 2.89, 3.12, and 3.29 Å resolution, respectively (Fig. 5G through I). The decrease in resolution at higher temperatures is expected as the addition of heat increases kinetic energy and disrupts intra-molecular bonding, resulting in destabilized proteins. Nonetheless, the resolution of 3.29 Å is sufficient to resolve the main chain, large side chains, and most additional side-chain densities. The model refinement statistics are provided in Table 1. The gross capsid morphology is conserved across the three temperatures. This includes an eight-stranded β-barrel core comprised of two four-stranded antiparallel beta sheets (βBIDG and βCHEF), an α-helix that forms the wall of the twofold depression, the DE loop which forms a turret around the fivefold axes, and the HI loop in the depression around the fivefold axes on the outside of the DE loop. The conformation of the variable regions is highly conserved at the three temperatures (Fig. 6D). These regions are termed variable based on differences between AAV serotypes (44), so the uniformity within AAV5 capsids at the different temperatures is expected. Notably, an extended N-terminus is observed at 80°C (Fig. 6F). To date, all AAV capsid structures reveal structural ordering of VP3 apart from approximately 15 amino acids of the N-terminal domain (NTD) (45). Surprisingly, incubation at 80°C increased ordering at VP3’s NTD, enabling the modeling of six additional residues leading up to A208 (A202, N203, N204, A205, Q206, and G207). This is unexpected given the well-established role of heat as destabilizing to protein structure. An increase in observed density at the NTD implies that the region has become more ordered at 80°C relative to 4°C and 55°C. The most apparent conformational change at 80°C is the loss of packaged genome (Fig. 5). This would imply that DNA destabilizes the NTD of VP3 or blocks stabilizing interactions that become available upon ejection. Interestingly, an extension of the observed NTD has not been described in conventionally purified empty AAV capsids—which are often used for structural analysis. These capsid populations are produced from capsids that failed to package the genome or were not transfected with a packaging plasmid. In this study, empty capsids are distinct in that they were once full capsids. During genome release, it is likely that these capsids underwent a conformational change not experienced in previously studied empty capsids.

**TABLE 1:** Cryo-EM data collection and model refinement statistics for AAV5 capsids at 4°C, 55°C, and 80°C

Parameter	AAV5 (4°C)	AAV5 (55°C)	AAV5 (80°C)
Micrographs	707	709	724
Electron dose (e^−^/Å^2^)	45	45	45
Pixel size (Å/pixel)	0.935	0.935	0.935
Particles used for the final map	23,000	42,158	44,589
Resolution (Å)	2.89	3.12	3.29
Model refinement statistics			
Map CC	0.8367	0.8282	0.8004
RMSD bond (Å)	0.010	0.010	0.010
RMSD angle (°)	0.959	0.964	0.925
All-atom clash score	6.76	7.27	9.16
Ramachandran (%)			
Favored	96.89	97.48	96.92
Allowed	3.11	2.52	3.08
Unfavored	0	0	0
Rotamer outliers (%)	0	0	0
C-β deviation	0	0	0

### Key features of AAV5 cryo-EM structure

**Fig 7:**
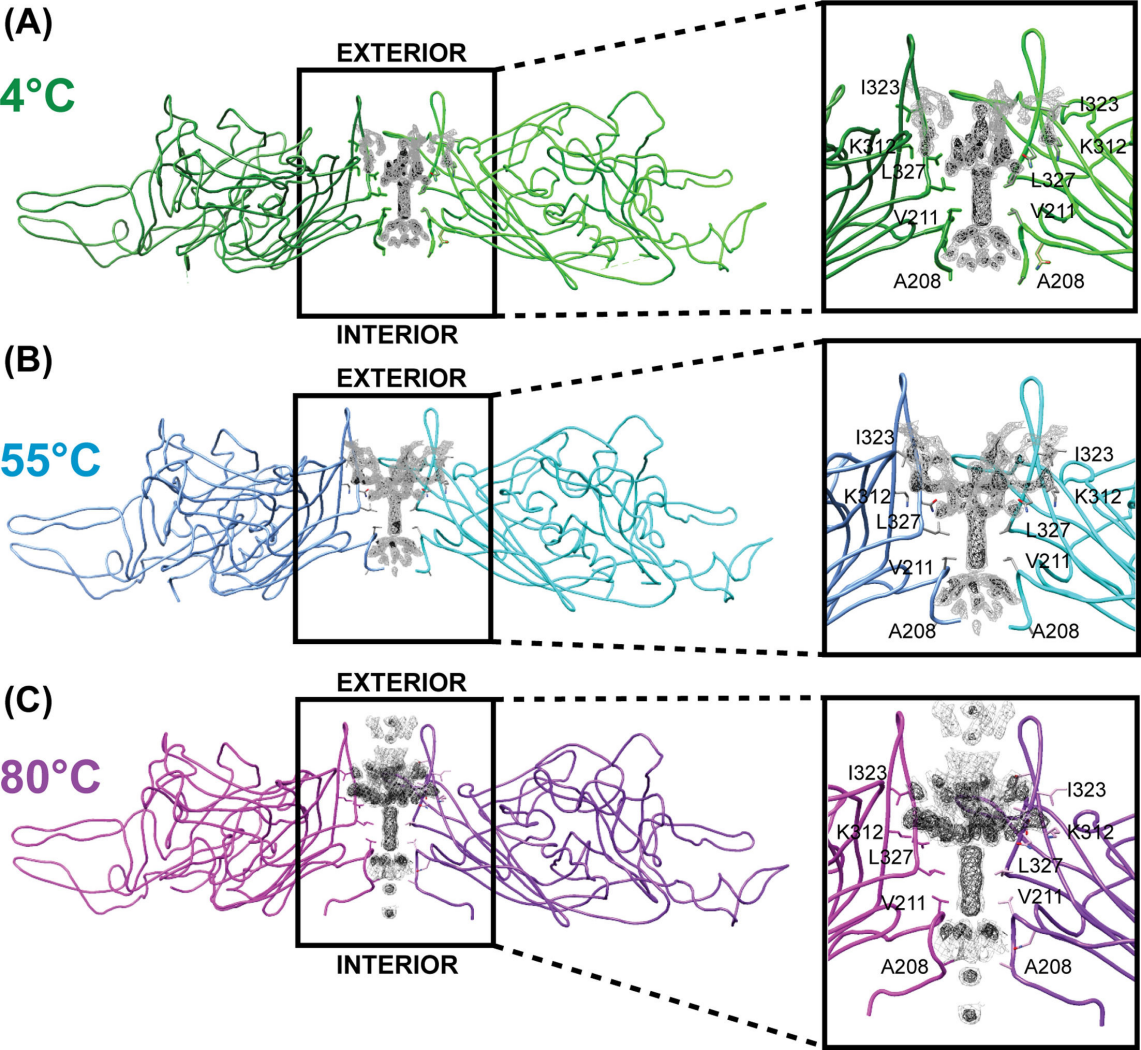
Density at the fivefold pore of AAV5 capsids. (**A**) AAV5 at 4°C (green), (**B**) 55°C (blue), (**C**) and 80°C (purple). Two opposing monomers of the pentamer are shown with a zoomed-in image of the fivefold pore shown to the right. Contour level 2 and level 1σ are shown in black and gray, respectively.

Five VP3 subunits are organized as pentamers to form the characteristic fivefold pore/channel. Sixty monomers in total form the assembled T = 1 icosahedral capsid, resulting in 12 fivefold pores per viral particle. A cross-sectional view of the AAV5 fivefold symmetry axis reveals rod-like density at the center of the fivefold channel. Interestingly the increasing incubation temperatures do not appear to impact this density’s core presence as it is maintained midway between the capsid interior and exterior (Fig. 7). The applied heat does impact the peripheral density surrounding the core rod. At 4°C and 55°C, density was observed between adjacent DE loops. Incubation at 80°C reveals additional, basket-like density at the capsid surface that is not visible at contour levels of 1σ for 4°C and 55°C (Fig. 7C). This density may exist in AAV5 capsids at 4°C and 55°C but is potentially too disordered to be observed. Though, it is unusual that increasing temperature would stabilize disordered interactions. It is possible then that this represents the movement of density toward the capsid exterior via the fivefold pore. The VP1u externalization is believed to occur through the fivefold pore, and VP1’s N-terminus has yet to be observed in any AAV structure to date. Previous identification of this density has been attributed to the N-terminal region of VP2 or the VP1/VP2 common region (46). There is a rod-like density that has also been observed in AAV1-VP3 capsids (45), suggesting VP1 and or VP2 do not exclusively occupy this space. In addition, the density is maintained in both full and empty capsids—confirmed here at 4°C and 80°C and in previous work (45, 46). Though, only full AAV8 and AAVrh.39 capsids have this described density (47). Collectively, these studies suggest that the density occupied in the fivefold pore is most likely the N-terminus of VP3, as suggested previously (48), and that this region may be stabilized by the presence of the viral genome and sensitive to changes in pH and temperature. It is important to note that icosahedral symmetry is imposed during 3D reconstruction. Icosahedral averaging does not account for potential heterogeneity at the symmetry axes. For example, it is not certain that all 12 fivefold pores are occupied by the same density, though this is assumed in the final structure. The NTD of VP3 may occupy most of these pores with a minor pore specialized for genome packaging, VP1u externalization, and GE. Potentially, VP3 functions as a “plug” at most of the fivefolds, directing the interior-exterior movement of proteins and genome to an alternative, select channel. A concentrated functionality at a single pore may enable more efficient capsid dynamics. Though, the evidence for this and the intrinsic determination of a specialized fivefold pore is speculative. In the future, local asymmetric reconstruction (49) or focused classification (50) will be used to better investigate the interior fivefold space.

**Fig 8:**
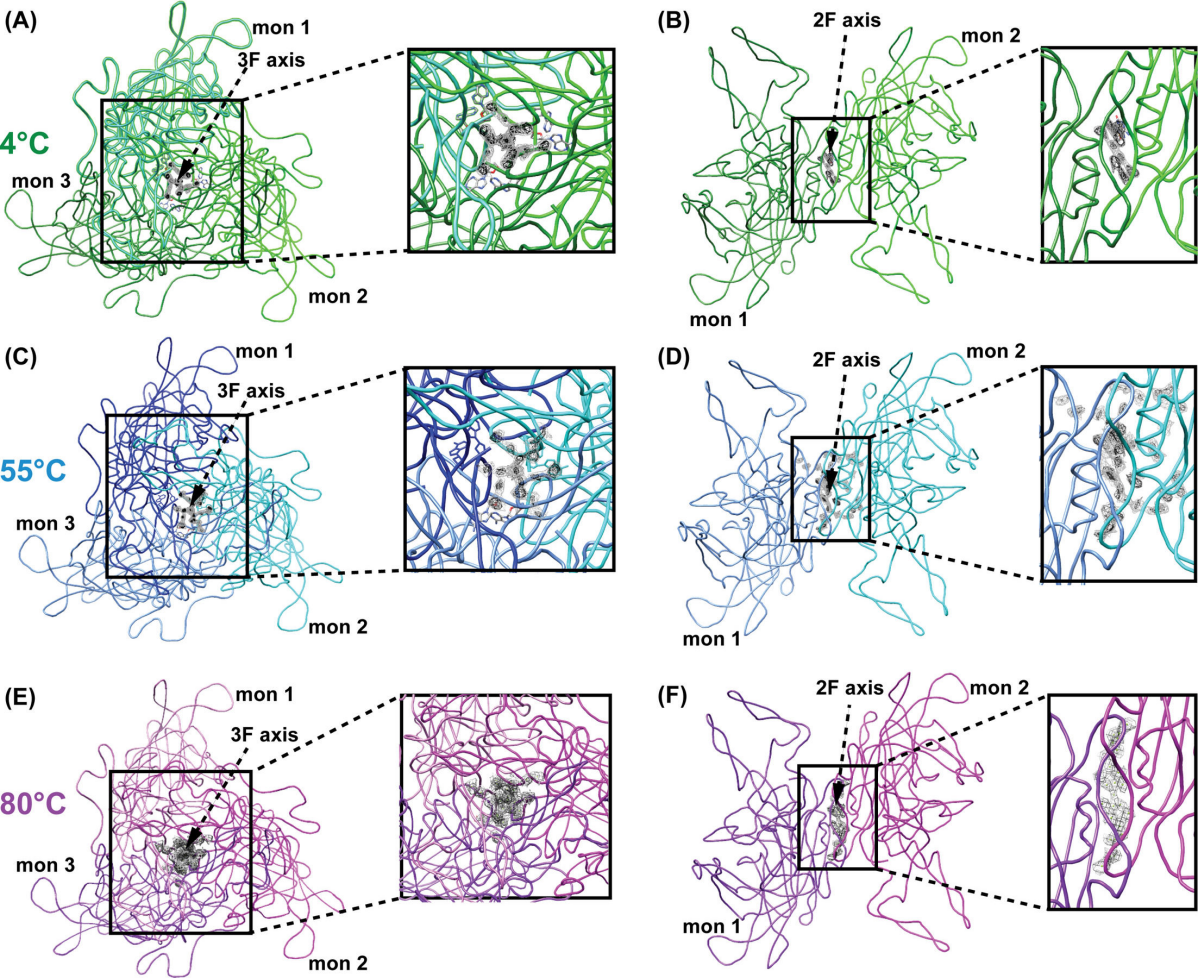
Density at the threefold and twofold symmetry axis of AAV5 capsids. (**A**) AAV5 trimer at 4°C (green), (**C**) 55°C (blue), (**E**) and 80°C (purple). An aerial view of the trimer is shown with a zoomed-in image of the threefold axis shown to the right. (**B**) AAV5 dimer at 4°C (green), (**D**) 55°C (blue), (**F**) and 80°C (purple). A dimer is shown with a zoomed-in image of the twofold axis shown to the right. Contour level 2 and level 1σ are shown in black and gray, respectively.

The protrusions at the threefold axis of the AAV capsid have been implicated in cell surface interactions and post-entry processing (51). In this study, there is particular interest in the DNA binding region under the threefold axis. At the center of the threefold, a basket-like density is observed at 4°C, 55°C, and 80°C (Fig. 8). The density extends outward with increasing temperature, but generally retains its shape in all three maps. The DNA binding pocket is situated below and adjacent to this density and is comprised of amino acids E417, V419, P420, D609, H630, P631, and S632 which have been shown to interact with a nucleotide between P420 and P631 (26). DNA is modeled in the AAV5 structures at 4°C and 55°C, but the density is not observed at 80°C (Fig. 8). This is consistent with the established characterization of predominantly empty capsids at 80°C (Fig. 5F). Residues H619, P620, S621, and P622 are adjacent to the modeled nucleotide at 4°C and 55°C (Fig. 8), providing similar side chains as the DNA binding region. Previously, a conserved residue P622 was investigated with AAV2 capsids, and it was shown that an alanine substitution resulted in a severely reduced genome packaging phenotype (25). The P622A variant was the most package-defective, or least effective at retaining the viral genome, of the nine threefold variants studied (25). The findings described here further support the established DNA binding pocket and reveal that this nucleotide retains its interior position at 55°C.

The twofold axis is characterized by a depression flanked by threefold protrusions of two distinct trimers. The twofold/fivefold wall is between the twofold depression and fivefold channel, and this interface is important for proper capsid assembly (25). Previous structural analysis of AAV capsids identified “globules” under the surface of the twofold axis that were attributed to the N-terminus of VP1 and VP2 (42, 52). This corresponds with the observation that tyrosine cross-linking at the twofold axis blocks VP1u externalization (19). An additional study demonstrated that twofold mutants had severely reduced infectivity (53). Interestingly, the mutants packaged and released normal levels of the viral genome but were defective for transcription, implicating an additional function unrelated to VP1u externalization (53). Collectively, the twofold axis joins the threefold and fivefold as a dynamic capsid region important for multiple viral processes. Additional density is observed at the interior side of the twofold axis at 4°C, 55°C, and 80°C. The density appears to be coordinated between the two dimers and is maintained with increasing temperature (Fig. 8). At 4°C, a nucleotide has been modeled at the twofold interface situated in between the unresolved density and residues T223, W224, M225, G226, R289, and L290 (Fig. 8). At 55°C and 80°C, the density no longer accommodates a nucleotide, though this may be attributed to disordered density and not a lack of DNA at 55°C. While density in the twofold has typically been associated with VPs, here we model a nucleotide at the twofold interface, that is not present when the genome is released from the capsid.

### Conclusion

The AAV capsid is multifunctional and plays an important role in the dynamics of the viral life cycle. The capsid serves a rudimentary function as a protective shield and transporter of the viral genome, and previous mutational analysis at the symmetry axes reveals additional roles in genome packaging and VP1u activity. In this study, we developed an assay to trigger GE and determined high-resolution cryo-EM structures of AAV5 capsids pre- and post-GE. The assay revealed that GE is serotype-independent, pH-sensitive, and dependent on VP1/VP2. It was hypothesized that incubation of AAV5 capsids at 80°C would induce the release of the viral genome and potentially reveal an avenue for ejection after comparative structural analysis with full AAV capsids. The loss of genome after heat treatment is quantified and paired with observed “string-like” material, likely DNA, emerging from intact capsids (Fig. 5). Subsequent changes in interior density are seen across the three states of GE (Fig. 7 and 8). While most of this density is unresolved, proximal VP3 residues have been identified as potential interactors and will be the focus of future structure-function investigations.

## MATERIALS AND METHODS

### *Sf9* production system

*Spodoptera frugiperda* insect (Sf9) cell lines containing AAV2 and AAV5 cap gene and AAV2 rep gene were grown in suspension culture using Sf-900 II Serum-Free Medium (Thermo Fisher) with 1% Antibiotic-Antimycotic (100X, Thermo Fisher) (54). The culture was incubated at 37°C and cells were passaged to 0.5 × 10^6^ cellular concentrations. Cell lines were infected with baculovirus packaging pTRUF-26 using a multiplicity of infection of 5. 72 h after infection, cells were harvested by centrifugation at 4,000 revolutions per minute (rpm) for 10 min in a JA-10 rotor. The cell pellet was resuspended in TD buffer (10 mM Na_2_HPO_4_, 2 mM KH2PO4, 135 mM NaCl, 5 mM KCl, 1 mM MgCl_2_, pH 7.4). Cells were lysed by three freeze-thaw cycles and treated with benzonase (0.1 µL/mL) for 1 h at 37°C. The supernatant was precipitated by 10% PEG8000 overnight and centrifuged at 9,000 rpm for 90 min in a JA-10 rotor. The supernatant was discarded, and the PEG pellet was resuspended in the TD buffer. The resuspended pellets were clarified by centrifugation at 9,000 rpm for 20 min in a JA-20 rotor, resuspended with 0.5 M NaCl in 1XTD, and re-clarified prior to additional purification.

### HEK293 production system

Human embryonic kidney (HEK293) cells were maintained in Dulbecco’s modified Eagle’s medium with 1% Antibiotic-Antimycotic (100X, Thermo Fisher) and 10% fetal bovine serum in a 15 cm^3^ petri dish at 37°C and 5% CO_2_. To produce wt and mutant recombinant adeno-associated viruses (rAAVs), a triple transfection was performed using a pXR construct (13 µg) containing the AAV2 *rep* gene and the *cap* gene of either AAV2 or AAV5, pHelper (20.5 µg) containing the adenovirus helper genes, and pTR-UF11-GFP (10 µg) containing the reporter gene and AAV2 inverted terminal repeats. Transfection was completed using polyethyleneimine and cells were incubated at 37°C for 72 h. Cells were harvested as described for the insect cell line.

### Iodixanol and column chromatography

When optimizing for genome-containing capsids, the clarified cell lysate was purified by a discontinuous iodixanol step gradient from a previously established protocol (31). The sample was examined for VPs using western blot and the genome was tittered using qPCR. Iodixanol fractions containing VPs and packaging genome were diluted 5× into Buffer A (20 mM Tris Base, 15 mM NaCl, pH 8.5), and loaded onto a 5 mL Hi-Trap Q Sepharose High Performance anion exchange column (Cytiva). The sample was eluted using a fast protein liquid chromatography system. Elution was done using an increasing gradient of Buffer B (20 mM Tris Base, 500 mM NaCl, pH 8.5), from 0% to 100% Buffer B in 30 min. 1 mL fractions were collected at a rate of 1 mL/min and analyzed using SDS-PAGE. Fractions with visible VPs were combined and concentrated using a 100 kDa molecular weight cut-off concentrator. The purified AAV2-fulls and AAV5-fulls were used as stock samples for the heat-treated virus visualized by negative stain EM and structure determination by cryo-EM.

For the pH and VP3-only experiments, the virus was purified by AVB column chromatography. The clarified cell lysates were diluted 1:5 in TD buffer and loaded onto a 1 mL AVB-Sepharose HP column (GE Healthcare) at a rate of 0.5 mL/min. The column was washed with 20 mL of TD buffer before elution with 10 mL elution buffer (0.1 M glycine-HCl, pH 2.7) into 1 mL of neutralization buffer (1 M Tris-HCl, pH 10). The sample was concentrated using a 100 kDa centrifugal filter unit and the buffer was exchanged using TD buffer. Capsid integrity and purity were verified with negative stain-EM, cryo-EM, and SDS-PAGE.

### SDS-PAGE

To determine sample purity and check for the presence of VPs, SDS-PAGE was used. A 2.5 µL of Laemmli SDS sample buffer reducing (6×) and 10 µL of the sample were combined and denatured by boiling at 100°C for 10 min in a dry bath heat block before loading onto a 10% polyacrylamide gel. The gel was run at 80 V in the stacking stage and increased to 130 V in the resolving stage. The gel was stained with GelCode Blue Safe Protein Stain for at least 1 h and de-stained with three washes of diH_2_O.

### Western blot

Western blot was performed to verify the presence of VPs in iodixanol fractions using a VP3 C-terminus specific antibody (mAb B1) prior to additional purification. The protocol for SDS-PAGE was followed through the completion of electrophoresis for the western blot. After initial electrophoresis, the gel was run at 0.15 A for 2 h in a semi-dry blotter to transfer the proteins to a nitrocellulose membrane soaked in western transfer buffer (25 mM Tris, 192 mM glycine, 20% vol/vol methanol). The membrane was blocked for 1 h in 5% wt/vol milk in 1× PBS + 0.1% Tween 20. The mouse IgG primary antibody mAb B1 was diluted 1:20,000 in 1% wt/vol milk in 1× PBS + 0.1% Tween 20 and incubated with the nitrocellulose membrane for 1 h. The membranes underwent three 5-min washes in 1× PBS + 0.1% Tween 20 before secondary antibody incubation. Anti-mouse horseradish peroxidase-conjugated secondary antibody (GE Healthcare) was diluted 1:10,000 in 1× PBS + 0.1% Tween 20 and incubated with the membranes for 1 h. The membranes were washed as described previously prior to development in a 1:1 solution of peroxide solution and luminol (ThermoFisher) with the signals detected on X-ray film.

### Differential scanning fluorimetry

DSF utilizes a sensitive protein stain, SYPRO orange, that fluoresces when bound to hydrophobic proteins. These residues are inaccessible when the capsid is assembled and become accessible upon unfolding and denaturing. A 2 µL of purified sample, 20.5 µL of a pH-specific (4, 5.5, 6, 7.4) universal buffer (20 mM HEPES, 20 mM MES, 20 mM NaAc, 150 mM NaCl) and 2.5 µL of 1% SYPRO orange (Invitrogen) were combined and incubated on ice for 5 min. An additional control for background fluorescent signal was used by combining buffer and SYPRO orange only. The assay was run using a Bio-Rad MyiQ2 thermocycler instrument, and the experimental temperature ranged from 30°C to 99°C with temperature ramping of 0.5°C per step. The rate of change of fluorescence with temperature was recorded as an inverse thermal profile, –dRFU/dT versus temperature. The –dRFU/dT values were multiplied by −1 and normalized to 1 by dividing the raw values with the peak value for evaluation. The peak value is recorded as the T_M_. All experiments were conducted in triplicates.

### Site-directed mutagenesis

To investigate the role of VP1/VP2 on genome ejection, constructs that only express VP3 were created using site-directed mutagenesis. Mutants were constructed using the Quikchange II site-directed mutagenesis kit (Agilent Technologies; Santa Clara, CA). Mutants were made in either a pXR2 (AAV2) or pXR5 (AAV5) background. PCR primers were designed with 18 or 15 nucleotides flanking the desired mutation for VP1 and VP2 mutagenesis, respectively. The mutants were confirmed by sequencing the *cap* ORF. Mutant viruses were prepared by polyethyleneimine transfection of HEK293 cells and purified as previously described. All viruses were packaged with pTR-UF11 containing a GFP expression cassette. The DNA titer was determined using qPCR in the Bio-Rad MyiQ2 thermocycler.

### Native immuno-dot blot

The cross-reactivity of AAV capsids after heat treatment with monoclonal antibodies against conformational epitopes of AAV serotypes 2 and 5 was tested using native immuno-dot blots. A 25 ng of virus (1 ng/µL) was applied to a nitrocellulose membrane using a dot blot manifold attached to a vacuum filtration system. Membranes were blocked for 1 h in 6% wt/vol milk in 1× PBS + 0.1% Tween 20. The mouse IgG primary antibodies were diluted 1:3,000 for HL2476, 1:300 for A20, 1:20,000 for B1, and 1:250 for A1 in 1% wt/vol milk in 1× PBS + 0.1% Tween 20 and incubated with the nitrocellulose membrane for 1 h. The membranes were treated as described for the western blot protocol.

### Quantitative polymerase chain reaction

To determine the packaged vector genome titer of purified samples, DNase treatment was first performed in a Bio-Rad MyiQ2 thermocycler at 37°C for 15 min. DNase was inactivated by a subsequent incubation at 95°C for 10 min. PCR was done using iQ SYBR Green Supermix using a CFX Connect PCR machine running the CFX manager software suite (Bio-Rad). Standards were loaded in technical duplicates from 10, 1, 10^−1^, 10^−2^, 10^−3^, 10^−4^, and 10^−5^ ng, alongside a non-template control using diH_2_O. The protocol was done as described for the genome ejection thermal assay, but samples were incubated for 30 min at the indicated temperatures prior to DNase treatment.

### Alkaline agarose gel electrophoresis

Alkaline agarose gel electrophoresis was used to visualize the loss of packaged genome from intact capsids after heating. A 1% agarose gel was prepared using TAE buffer (40 mM Tris-Acetate, 1 mM EDTA) and incubated in denaturing buffer (50 mM NaOH, 1 mM EDTA) for at least 3 h. Samples (at 2 × 10^10^ vg) were loaded with 2× denaturing loading dye. Electrophoresis was done at 40 V for 14–16 h at 4°C. The gel was washed with TAE buffer for 30–60 min on the shaker before being stained with SYBR Gold staining solution (30 µL SYBR Gold 10,000×, 175 mL TAE buffer) for an additional 30–60 min. The gel was imaged using a fluorescent gel scanning unit.

### Negative stain transmission electron microscopy

To determine the gross morphology of the capsids and characterize the population of genome-containing particles, 5 µL purified virus was loaded onto a glow-discharged carbon-coated copper EM grid (Ted Pella, Inc., cat. no. 01754-f) for 2 min and negatively stained with 5 µL of 2% uranyl acetate for 20 s. The grids were visualized on a Tecnai G2 Spirit TWIN device equipped with a charge-coupled device camera at a magnification of 40,000×.

### Cryo-electron microscopy data collection

After 30 min temperature incubations, 3 μL of the AAV5-fulls (stock sample) were applied to glow-discharged C-flat holey carbon-coated grids (Protochips Inc.) and vitrified using the Vitrobot Mark IV (FEI) automatic plunge-freezing system. The sample was incubated on the grids at 4°C and 95% humidity for 3.0 s prior to blotting using filter paper and plunging into an ethane slush, cooled with liquid nitrogen, for vitrification. The grids were maintained at liquid nitrogen temperatures until data collection. The particle distribution and ice quality of the grids were screened in-house using a FEI Tecnai G2 F20-TWIN microscope (FEI Co.) operated under low-dose conditions (200 kV, ~20e−/Å2). The data collection for high-resolution cryo-EM was performed at Stanford University’s Linear Accelerator Center (SLAC) Cryo-EM Center using the Titan Krios electron microscope. Information regarding data collection is described in Table 1.

### 3D image reconstruction

For the three-dimensional image reconstruction, the cryoSPARC software package was utilized (43). The aligned micrographs were imported into the program and the patch contrast transfer function estimation was used to estimate defocus variation in the sample. This was followed by automatic blob picking with a minimum particle diameter of 240 Å and a maximum particle diameter of 260 Å. The particles were quality inspected and selected particles were accepted for further refinement. The accepted particles were extracted with an extraction box size of 400 pixels. The extracted particles were subjected to an automatic 2D classification followed by the manual selection of final 2D classes. A template was generated based on the 2D classes and used to pick particles from the original, aligned micrographs. These particles were inspected, extracted, and organized into 2D classes as described previously. From the selected 2D classes, *ab initio* was performed to generate a 3D map followed by a homogenous refinement using icosahedral symmetry. Three sharpened maps were generated with a B factor value of ±20 from the Guinier Plot. The resolutions of the reconstructed maps were estimated based on a Fourier Shell Correlation of 0.143.

### Model building

A 60mer was generated for AAV5 at 4°C, 55°C, and 80°C using the oligomer generator function in ViperDB (55). The 60mer models were positioned into the cryo-EM density maps using the “fit in map” tool in Chimera (56). The pixel size was adjusted to maximize the correlation coefficient between the maps and the models. The EMAN2 subroutine “e2proc3D.py” was then used to convert maps to the Xplor map format (57), and MAPMAN was used to convert from the Xplor map to CCP4 format (58). Model refinement of the monomers was initiated in Coot using the real space refinement tool (59), with manual modifications made as necessary. Monomer representations with labeled secondary structures, symmetry axes, surface loops, and the C- and N-terminal domains (CTD, NTD) were generated in pyMOL (60). Automatic refinement of the 60mer was then performed using PHENIX (61). The final model statistics are shown in Table 1. The Cα atoms of the final refined VP3 structure for AAV5 at 4°C, 55°C, and 80°C were superposed using the secondary structure matching superpose option in Coot (59), enabling comparison of the main and side chains. The side-chain density images were generated with the UCSF-Chimera program (56).

## Data Availability

The AAV5 cryo-EM reconstructed density maps and models built for the capsids at different temperatures (4°C, 55°C, and 80°C) were deposited in the Electron Microscopy Data Bank (EMDB; https://www.ebi.ac.uk/emdb/) with accession numbers EMD-46748 Protein Data Bank (PDB) ID 9DCB (4°C), EMD-46749 PDB ID 9DCC (55°C), EMD-46745, and PDB ID 9DC7.
